# Efficacy and safety of ledipasvir/sofosbuvir for hepatitis C among drug users: a systematic review and meta-analysis

**DOI:** 10.1186/s12985-021-01625-w

**Published:** 2021-07-27

**Authors:** Xue Yang, Yang Tang, Di Xu, Guang Zhang, Peng Xu, Houlin Tang, Lin Pang

**Affiliations:** National Center for AIDS/STD Control and Prevention, Chinese Center for Disease Control and Prevention, Beijing, China

**Keywords:** Hepatitis C, Ledipasvir and sofosbuvir, Drug users, SVR12, Meta-analysis

## Abstract

**Background and aims:**

Limited data is available on the efficacy of direct acting anti-viral drugs on hepatitis C in drug users. The aim of this meta-analysis was to comprehensively analyze the efficacy and safety of LDV/SOF in drug users infected with the hepatitis C virus (HCV).

**Methods:**

The PubMed, Cochrane library, Embase and Web of Science databases were searched for articles published till April 2021 on HCV-positive drug users who were treated with ledipasvir/sofosbuvir (LDV/SOF). The primary endpoint was pooled sustained virological response at 12 weeks (SVR12) with 95% confidence interval (95% CI). Funnel plots and Egger’s test were used to assess the publication bias.

**Results:**

A total of 12 studies and 711 subjects treated with LDV/SOF-based regimen for HCV were included, and the pooled SVR12 rate was 89.8% (95% CI 85.9–92.7). The pooled SVR12 rate of genotype 1 drug users was 92.4% (95% CI 88.6–95.0). Subgroup analysis showed that pooled SVR12 rates of patients treated with LDV/SOF and LDV/SOF ± RBV were 89.2% (95% CI 83.4–93.1), 90.4% (95% CI 83.6–94.5) respectively. In addition, the SVR12 rates were 88% (95% CI 70.7–95.7) for 8 weeks, 89.9% (95% CI 81.0–94.9) for 12 weeks and 82.2% (95% CI 24.9–98.5) for 24 weeks of treatment.

**Conclusion:**

LDV/SOF is a safe and relatively effective treatment for hepatitis C in drug users.

**Supplementary Information:**

The online version contains supplementary material available at 10.1186/s12985-021-01625-w.

## Introduction

Chronic hepatitis C can lead to liver fibrosis, which eventually progresses to cirrhosis and increases the risk of primary liver cancer [1]. Although the World Health Organization (WHO) has set a goal to eliminate hepatitis C virus (HCV) globally by 2030, its 2017 Global Hepatitis Report shows that 71.1 million people were chronically infected with HCV by 2015 [2, 3]. This goal has been achieved in Iceland, but is challenging to tackle in the United States and sub-Saharan Africa since the HCV epidemic in these regions is related to intravenous drug use [4]. The prevalence of HCV infection among drug users ranges from 30 to 70% [5].

Furthermore, even a considerable burden of HCV infection does not prevent drug use among the long-term abusers [6]. The HCV load is significantly increased in people who inject drugs (PWID), and drug use accounts for 23% of the newly identified infections [2, 7]. Furthermore, prolonged drug use in the absence of suitable interventions can significantly increase the risk of HCV infection [8].

Many PWID with chronic hepatitis C could not be treated in the past due to concerns regarding their alcohol consumption, pre-existing psychiatric disorder, and intravenous drug use [9]. Since 2014, direct-acting antiviral (DAA) drugs have completely revolutionized the treatment of chronic HCV infection, and the combination of 2–3 DAAs achieved sustained virological response (SVR) in more than 95% of the treated patients without requiring any interferons [10]. Ledipasvir/sofosbuvir (LDV/SOF) is the first DAA combination approved by the American Association for the Study of Liver Diseases (AASLD) and the American Infectious Disease Society for treating HCV genotype 1 [11]. Moreover, the fixed dose combination of the NS5B polymerase inhibitor SOF and the NS5A inhibitor LDV has marked a new era for patients with chronic HCV with genotype la, 1b, and 4 because it is the first drug to be approved by the FDA that does not include peginterferon (PEG) or ribavirin (RBV) [12]. The Asian-Pacific Association for the Study of the Liver (APASL) guidelines advices Ledipasvir (90 mg/day) with sofosbuvir (400 mg/day) for 12 weeks is also associated with high SVR12 rate in HCV genotype 2 infected patients including treatment-experienced and those with cirrhosis [13]. In according to the European Association For The Study Of The Liver(EASL) guidelines, all people who inject drugs (PWIDs) who are infected with HCV, including those receiving OST, those with a history of injecting drug use and those who recently injected drugs, should be treated with the general recommendations [14].

LDV/SOF with or without RBV was the most frequently used medication regimen [15]. Now, none of the guidelines specify a priority treatment plan for HCV treatment for drug users. Whether LDV/SOF has advantages in treating drug users is a question worthy of research.

The poor compliance of chronic drug users is a persistent barrier to treating HCV. In addition, the high cost of DAA drugs and the risk of re-infection further limits the benefits of this treatment regimen among drug users [16]. There are relatively few studies on the effects of antiviral treatment in this population, and fewer on the efficacy of LDV/SOF regimens. Therefore, the aim of this study is to systematically review the studies evaluating the efficacy and safety of LDV/SOF in achieving SVR12 in drug users with HCV.

## Methods

### Literature search strategy

The study was conducted according to the preferred reporting items for systematic review and meta-analysis (PRISMA) protocols [17]. The Pubmed, Cochrane library, Embase and Web of Science database were searched independently by two investigators (X.Y. and T.Y.) for articles published till April 2021 using the following MeSH terms: “Hepatitis C” (e.g. “HCV”); “Drug User” (e.g. “People who inject drugs”, “IDUS”); “ledipasvir, sofosbuvir drug combination” (e.g. “Harvoni”). The complete literature search strategy for the four databases can be found in Additional file 1. The bibliographies of the selected articles were also searched manually for additional studies. The clinical trial registry for additional trials was also checked. There were no filters for language or publication date.

### Study selection

**Fig. 1:**
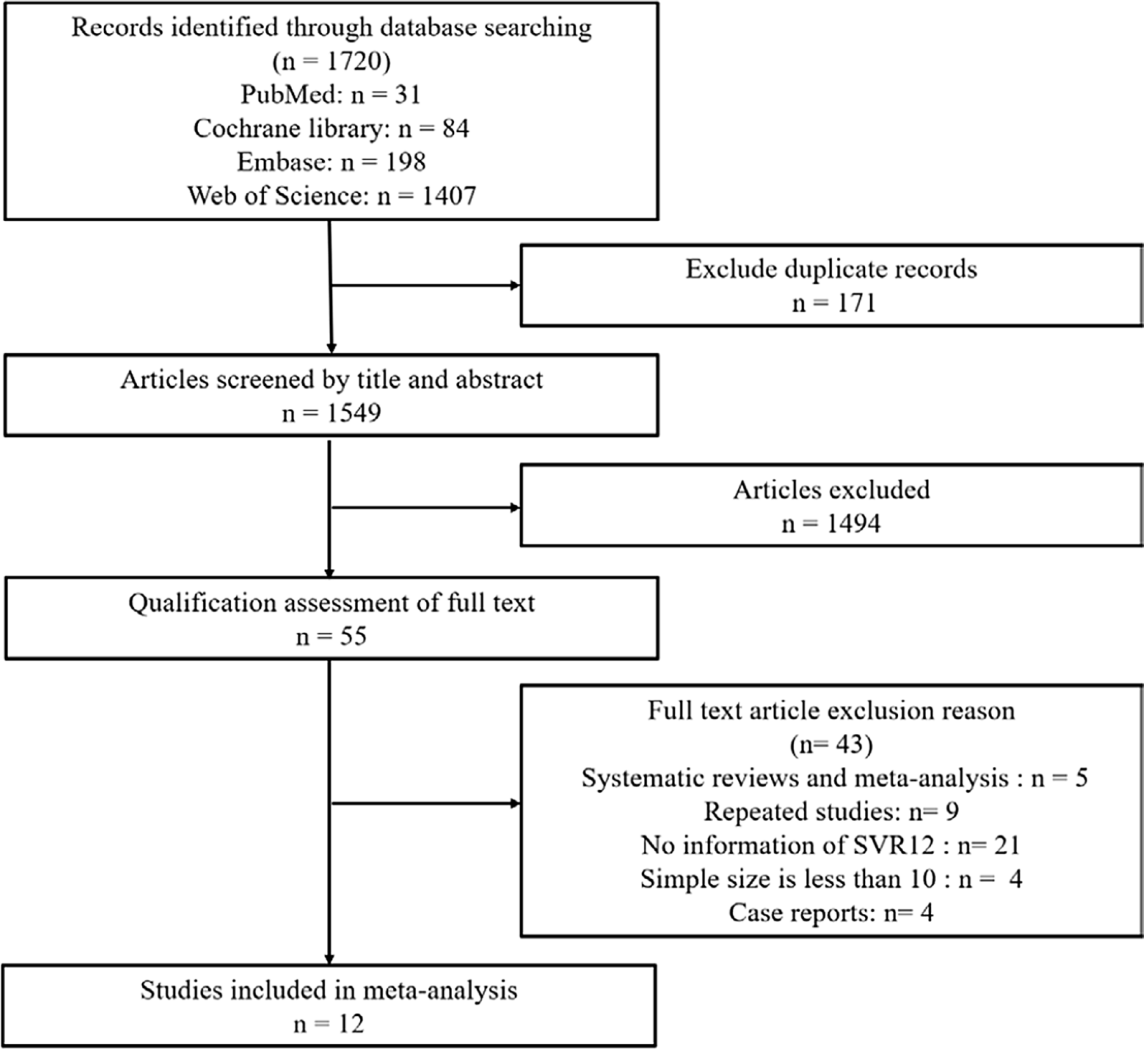
The flow diagram of literature search according to PRISMA

The titles and abstracts were independently screened by two investigators (X.Y. and T.Y.), and the eligible studies were additionally validated by a third investigator (X.D.). The inclusion criteria were as follows: (1) study subjects were hepatitis C patients who use or inject drugs, (2) ledipasvir + sofosbuvir intervention, and (3) clear SVR12 as the outcome. The exclusion criteria and the number of excluded studies were shown in Fig. 1.

### Data extraction

The following data was extracted by the two primary investigators using a standard form: (1) study characteristics (writer, region, publication year, study period, study design, setting), (2) drug use characteristics (definition of recent drug use; OST, OAT), (3) treatment characteristics (medications, treatment duration, drug dose, HCV treatment experience), (4) patient characteristics (genotype, sex, age, weight, BMI, race, education, aboriginals, homelessness/unstable housing, employment status, relationship status, involvement in sex work, imprisonment record, annual Income, alcohol overuse, HIV co-infection, HBV co-infection, cirrhosis, prior history of HCC, adherence support), and (5) outcome characteristics (number of SVR12, relapse, reinfection, virological failure, adverse events). The inconsistencies were then confirmed by the third investigator. Since most of the included literature was concerned with LDV/SOF and other drugs, we initially extracted all drug information to obtain complete baseline data. Some relevant information was obtained from ClinicalTrials.com via the NCT numbers.

### Quality assessment

The quality of the observational studies was assessed with the Newcastle–Ottawa quality assessment scale (NOS). The studies were scored on the basis of three aspects: selection (4 points in total), comparability (2 points in total) and outcome of study participants (3 points in total). Total score of less than 5 was considered low quality, 6–7 as moderate quality, and greater than 8 as high quality [18]. The Cochrane Collaboration’s tool was used to assess the risk of bias in the randomized controlled trials (RCTs). Seven domains were evaluated: (1) random sequence generation (selection bias), (2) allocation concealment (selection bias), (3) blinding of participants and personnel (performance bias), (4) blinding of outcome assessment (detection bias), (5) incomplete outcome data (attrition bias), (6) selective reporting (reporting bias), and (7) other bias. Each domain was assessed separately with three options: “Low risk”, “Unclear risk” and “High risk” [19]. Two investigators performed the primary assessment, and the third verified and summarized the results.

### Outcomes

The primary outcome was the proportion of patients who achieved a sustained virological response (SVR) 12 weeks after discontinuation of treatment (SVR12). The secondary outcomes were as follows: (1) relapse—recurrence of HCV RNA within the post therapy follow-up period [20], (2) reinfection—detection of HCV RNA following end of treatment response or following sustained virologic response [21], (3) virological failure—increase in the HCV RNA level to at least 100 IU/ml from < 15 IU/ml during treatment, by > 1 log_10_ IU/ml from the lowest levels attained during treatment, or to at least 15 IU/ml after 6 weeks of treatment [22], and (4) adverse events (AEs; any grade. e.g. fatigue, headache, nausea), severe adverse events (SAEs. e.g. death) and discontinuation due to AEs.

### Statistical analyses

The outcomes of proportion were pooled using the Wilson score method, and 95% confidence intervals (95% CIs) were used to compare the safety and efficacy of the pooled SVR12 rate with the inverse variance method. Heterogeneity across the included studies was assessed with Cochran Q-statistics and I^2^ statistics. The random effects model was used in case of significant heterogeneity (*P* < 0.10 and I^2^ > 50%), otherwise the fixed effects model was adopted. To further evaluate the efficacy and safety of DAA regimes, SVR12 was analyzed in subgroups stratified on the basis of genotypes, sex, presence/absence of intravenous drug use, HCV treatment experience, presence/absence of OST, HIV co-infection, alcohol overuse, homelessness or unstable housing, employment status, and relationship status. Likewise, the efficacy and safety of LDV/SOF were evaluated in the different treatment regimen and treatment duration subgroups. Publication bias was analyzed with funnel plots and Egger’s test. All the statistical tests were two-sided, and *P* value < 0.05 was considered statistically significant. All analyses were conducted using the Meta package in R (4.0.2).

## Results

### Search results and study characteristics

We searched a total of 1720 articles from four databases. After removing the duplicate entries, 1549 articles were scanned further and 55 were selected after excluding those with irrelevant titles and abstracts. After reading the complete articles, 43 were excluded for various reasons and 12 (11 full-length articles and 1 abstract) [23–34] were included for the final analysis (Fig. 1). Given the peculiarities of drug users, the sample size in each study was relatively small. All included articles were published after 2016. The studies were either observational studies or RCTs. In every study population, the males outnumbered the females. The patients were treated with LDV/SOF for 8, 12 and 24 weeks. The characteristics of the studies and drug users were summarized in Tables 1 and2. The Prisma checklist of this study was shown in the Additional file 4.

**Table 1:** Main characteristics of the studies included in meta-analysis

Study	Study design	Study period	Publication type	Region	Setting	Regimen	Duration (weeks)	Daily dose of LDV/SOF
Akiyama et al. [33]	Randomized trial	NA	Abstract	Sub-Saharan Africa	Multi-center	LDV/SOF	NA	NA
Akiyama et al.[34]	Randomized trial	2013.10–2017.4	Full-length	New York	Multi-center	LDV/SOF	4/8/12	NA
Gayam et al. [23]	Retrospective cohort study	2016.1–2017.12	Full-length	America	Multi-center	LDV/SOF ± RBV	NA	NA
Alimohammadi et al. [24]	Retrospective study	2015.9–2019.2	Full-length	Canada	Multi-center	LDV/SOF	NA	NA
Coffin et al. [25]	Two-arm randomized trial	2015–2017	Full-length	San Francisco	Single-center	LDV/SOF	8	80/400 mg
Schütz et al.[26]	Observational study	2015.9–2016.9	Full-length	Austria	Multi-center	LDV/SOF	8	90/400 mg
Øvrehus et al.[27]	Randomized trial	2015.4–2016.4	Full-length	Denmark	Single-center	LDV/SOF/RBV ± PEG	4	90/400 mg
Trabut et al. [28]	Retrospective case–control study	NA	Full-length	French	Multi-center	LDV/SOF ± RBV	8/12	NA
Grebely et al.[29]	Randomized trial	2012.10–2016.5	Full-length	6 Study locations	Multi-center	LDV/SOF ± RBV	8/12/24	90/400 mg
Morris et al. [30]	Observational study	2016.3–2017.2	Full-length	Australia	Single-center	LDV/SOF	8/12/24	NA
Read et al. [31]	Observational cohort study	2015–2017	Full-length	Australia	Single-center	LDV/SOF	8/12/24	NA
Grebely et al. [32]	Randomized trial	2013.11–2014.4	Full-length	88 study locations	Multi-center	LDV/SOF ± RBV	8/12/24	90/400 mg

**Table 2:** Patient characteristics of the studies included in meta-analysis

Study	Regimen	SVR12 (n/N)	Definition of recent drug use (measurement method)	OAT/OST	Age (year)	Sex (M/F)	Cirrhosis	HCV genotype	Treatment history	HIV/HBV coinfection
Akiyama et al. [33]	LDV/SOF	82/90	NA	NA	NA	NA	NA	GT 1,4	NA	NA
Akiyama et al.[34]	LDV/SOF	98/104	Drug use in the past or during DAA therapy (urine analysis and self-report)	OAT:150	51.2 ± 11.0	97/53	41	GT 1	16	21
	SOF/SMV	11/11								
	SOF/RBV	14/15								
	SOF/IFN/RBV	15/17								
	TVR/IFN/RBV	3/3								
Gayam et al. [23]	LDV/SOF ± RBV	94/101	Drug use during DAA therapy (urine analysis and self-report)	OAT:58	61.0 ± 9.8	93 /59	53	GT 1–4	29	44/NA
	OBV/PTV/r + DSV ± RBV	28/29								
	SOF + VEL ± RBV	20/22								
Alimohammadi et al. [24]	LDV/SOF	62/68	Drug use during the past 6 months (urine analysis)	OST:23	55	56/18	15	GT 1–3	NA	12/NA
Coffin et al. [25]	LDV/SOF	28/31	Actively injecting drug use in the past or during DAA therapy (urine analysis)	NA	42.43 ± 11.9	25/6	NA	GT 1	0	0/0
Schütz et al.[26]	LDV/SOF	40/40	Intravenous drug in the past or during DAA therapy (NR)	OAT:40	38.9 ± 8.7	29/11	0	GT 1	0	0/0
Ovrehus et al.[27]	LDV/SOF/RBV	12/16	Drug use in the past or during DAA therapy (self-report)	NA	− PEG:39.2(29.2–46.1)	− PEG:11/5	0	GT 1–3	0	NA/NA
	LDV/SOF/RBV + PEG	15/16			+ PEG:39.6 (26.4–48.2)	+ PEG:14/2				
Trabut et al. [28]	LDV/SOF	2/3	Drug use in the past or during DAA therapy (NR)	OST:34	46.2 ± 7.3	42/8	28	GT 1–4,6	12	4/1
	LDV/SOF ± RBV	17/19								
	SOF/DCV	25/27								
	SOF/SMV	1/1								
Grebely et al.[29]	LDV/SOF ± RBV	49/53	Drug use 12 months ago (urine analysis)	OST:194	48 ± 10.7	141/53	70	GT 1–6	42	NA/NA
	SOF + VEL	87/92								
	SOF/VEL/VOX	47/49								
Morris et al. [30]	LDV/SOF	49/62	Drug use in the past or during DAA therapy	OST:45	45.2 ± 10.5	82 / 38	NA	GT 1–3	10	NA/NA
	SOF/DCV ± RBV	41/50								
	OBV/PTV/r ± RBV	8/8								
Read et al.[31]	LDV/SOF	30/38	Injecting drug use in the past or during DAA therapy (urine analysis and self-report)	OST:18	45 (25–69)	48/23 Transgender:1	NA	GT 1–3	6	8/0
	SOF/DCV	23/28								
	OBV/DCV/ PTV/r ± RBV	6/6								
Grebely et al. [32]	LDV/SOF	45/48	Drug use 12 months ago (urine analysis)	OST:70	47 ± 11	48/22	7	GT 1	8	NA/NA
	LDV/SOF + RBV	21/22								

### Quality of the included studies

Six observational articles were assessed by NOS, and showed moderate quality with an average score of 7. Two studies were of high quality and three of medium quality. Six RCTs were assessed by the Cochrane Collaboration’s tool. None of the trials were conducted in a double-blinded manner, and only two mentioned random assignment, indicating higher risk of bias. The quality assessment of the included studies was summarized in the Additional file 2/Table 1, 2 and 3.

**Table 3:** SVR12 by settings; genotypes; sex; the presence or absence of intravenous drug use; HCV treatment experienced; the presence or absence of OST, HIV co-infected, alcohol overuse; homeless or unstably housed; the presence or absence of employed; and living in stable relationship

Response	SVR12 (N = 1069)		Heterogeneity		*P*^b^ value	Studies
	Total, n/N	Rate (95% CI)	*I*^2^ (%)	*P*^a^		
Overall	973/1069	90.6 (87.1–93.3)	59.9	0.0040		12
By settings						
Multi-center	761/814	93.1 (91.1–94.7)	0.0	< 0.0001	0.0040	8
Single-center	212/255	82.9 (77.7–87.1)	0.0			4
By genotypes						
1	512/549	92.4 (88.7–95.0)	32.3	0.1638	0.2163	8
2	15/15	90.0 (61.6–98.0)	0.0			3
3	36/44	80.9 (65.8–90.3)	0.0			3
4	6/7	81.0 (41.8–96.2)	0.0			2
6	1/1	75.0 (10.9–98.7)	–			1
By sex						
Male	260/294	88.6 (79.5–94.0)	64.8	0.1247	0.8051	5
FEMALE	123/139	87.4 (80.5–92.1)	0.0			5
By the presence or absence of intravenous drug use						
Yes	174/197	88.1 (78.1–93.9)	55.0	0.0214	0.8787	4
No	165/187	89.1 (74.9–95.7)	69.3			4
By HCV treatment experienced						
Naïve	205/227	90.2 (78.8–95.8)	72.0	0.1741	0.6157	3
Experienced	41/47	86.9 (73.8–94.0)	0.0			3
By the presence or absence of OST						
Yes	83/97	85.0 (76.2–90.9)	0.0	0.8280	0.5122	3
No	103/126	81.6 (73.8–87.5)	0.0			3
By the presence or absence of HIV co-infected						
HIV co-infected	49/56	86.8 (75.3–93.4)	0.0	0.2988	0.2620	3
Non HIV co-infected	145/154	93.5 (82.3–97.8)	63.7			2
By the presence or absence of alcohol overuse						
Overuse	85/91	92.7 (84.6–96.7)	0.0	0.1705	0.0492	3
No overuse	71/86	82.2 (72.5–90.0)	0.0			2
By homeless or unstably housed						
Homeless or unstably housed	52/61	91.1 (64.8–98.3)	39.7	0.0450	0.6915	2
Stable housing	45/49	86.4 (55.1–97.1)	70.0			3
By the presence or absence of employed						
Employed	14/16	83.9 (57.1–95.3)	0.0	0.1438	0.5555	2
Unemployed	116/131	84.5 (75.9–90.4)	68.5			3
By living in stable relationship						
Single	57/61	92.9 (69.4–98.7)	41.6	0.5195	0.7838	2
In a relationship	28/29	94.8 (80.0–99.0)	0.0			2

^a^Test of heterogeneity

^b^Test for subgroup differences

### Efficacy of outcomes and SVR12 rate

**Fig. 2:**
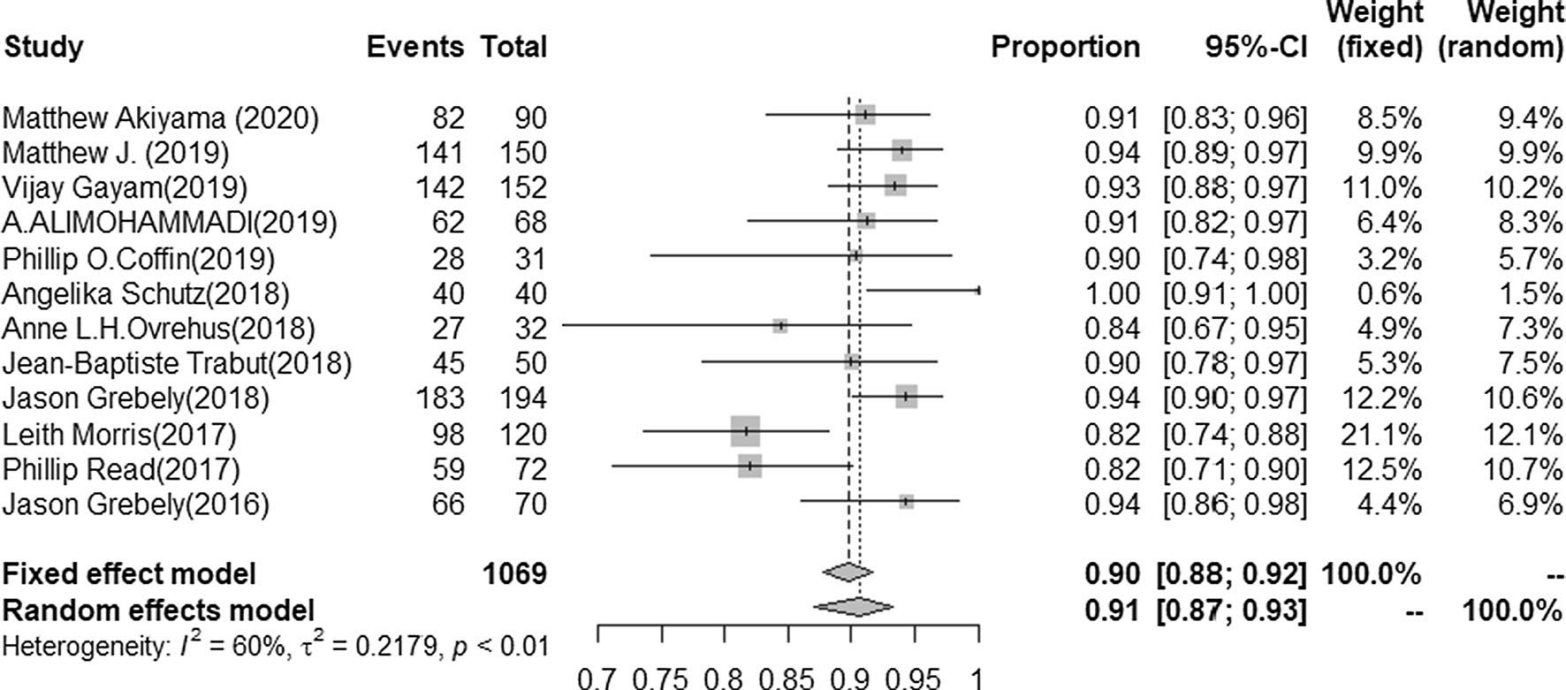
Forest plots of risk ratios and risk differences of SVR12 in all included literatures

**Fig. 3:**
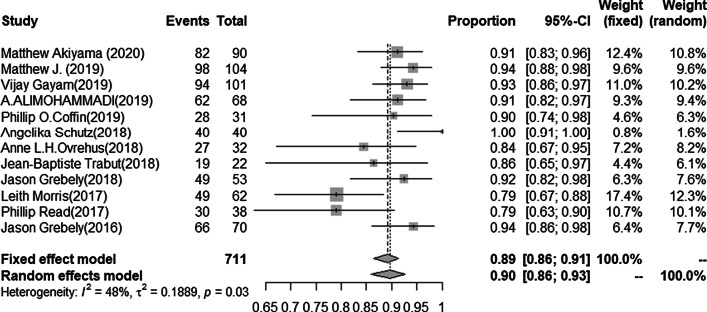
Forest plots of risk ratios and risk differences of SVR12 for LDV/SOF in all included literatures

**Fig. 4:**
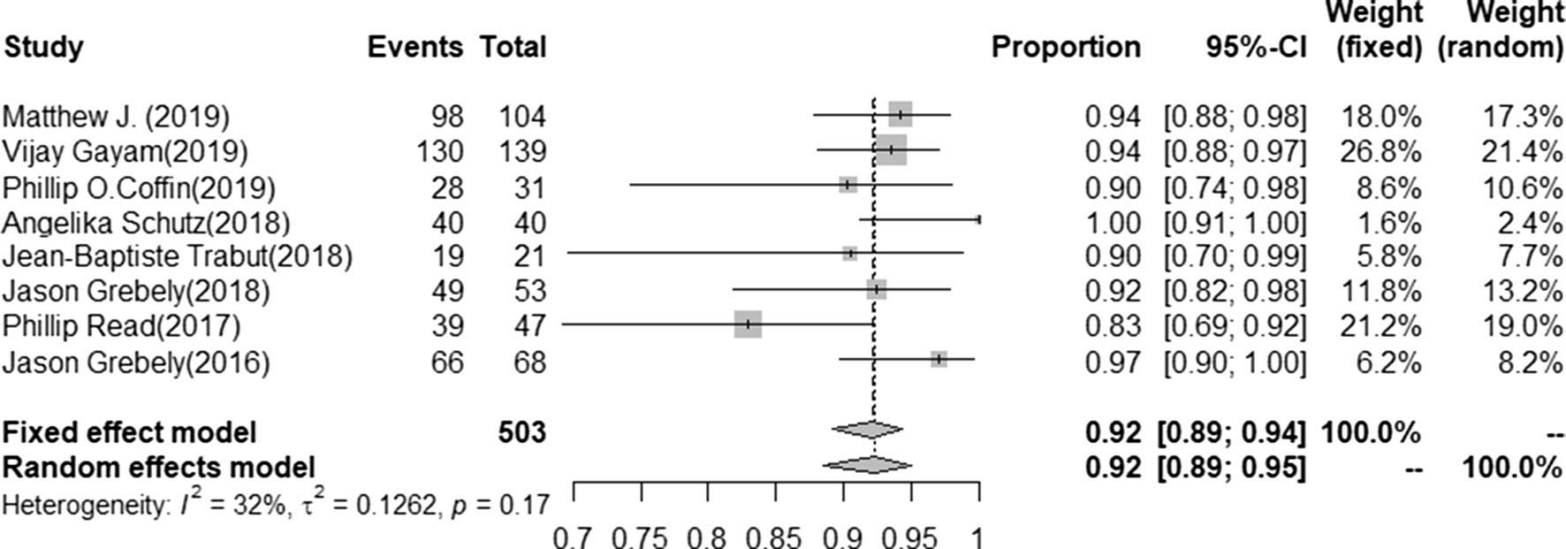
Forest plot of the risk ratio and risk difference of SVR12 in patients with genotype 1 using LDV/SOF in the included literature

The SVR12 rate of HCV infection was available for 1069 cases. The pooled estimated SVR12 rate from random-effects model was 90.6% (95%CI 87.1–93.3, I^2^ = 59.9%, *P* < 0.01) (Fig. 2). No publication bias was found in studies (t = 1.6.3 *P* = 0.13), and the funnel plot and Egger’s test results were shown in the Additional file 3/Figs. 1and2. The SVR12 rate of LDV/SOF regimen was available for 711 cases. The pooled estimated SVR12 rate from the random-effects model was 89.8% (95%CI 85.9–92.7, I^2^ = 47.6%, *P* = 0.03) (Fig. 3). No publication bias was found in studies (t = 1.78, *P* = 0.11), and the funnel plot and Egger’s test results were shown in the Additional file 3/Figs. 3and4. In eightstudies [23, 25, 26, 28, 29, 31, 32, 34] with a total of 503 patients with HCV genotype 1, 469 patients achieved SVR12 after LDV/SOF treatment. The pooled estimation of SVR12 rate from random-effects model was 92.4% (95% CI 88.6–95.0, I^2^ = 31.7%, *P* = 0.17) (Fig. 4).

### Subgroup analysis of SVR12 rate

As shown in Table 3, the pooled SVR12 rate was significantly higher in the multi-center (93.1%, 95% CI 91.1–94.7) compared to the single-center (82.9%, 95% CI 77.7–87.1) cohorts (*P* < 0.01). In terms of the HCV genotype, the pooled SVR12 rates for GT1, GT2, GT3, GT4 and GT6 were 92.4% (95% CI 88.7–95.0), 90% (95% CI 61.6–98.0), 80.9% (95% CI 65.8–90.3), 81% (95% CI 41.8–96.2) and 75% (95% CI 10.9–98.7) respectively. The pooled rates of SVR12 were similar between males and females [88.6% (95% CI 79.5–94.0) vs 87.4% (95% CI 80.5–92.1)], as well as between the intravenous and non-intravenous users [88.1% (95% CI 78.1–93.9) vs 89.1% (95% CI 74.9–95.7)]. In addition, 86.9% (95% CI 73.8–94.0) of HCV treatment-experienced patients achieved SVR12 compared to 90.2% (95% CI 78.8–95.8) of patients without prior HCV treatment. Pooled SVR12 rates were 85.0% (95% CI 76.2–90.9) for patients with OST and 81.6% (95% CI 73.8–87.5) for those without OST. The pooled rate of SVR12 was lower among patients with HIV co-infection [86.8% (95% CI 75.3–93.4)] compared to the non-HIV subgroup [93.5% (95% CI 82.3–97.8)]. SVR12 was achieved in 92.7% (95% CI 84.6–96.7) of patients with alcohol abuse compared to only 82.2% (95% CI 72.5–90.0) of the patients without alcohol abuse. Furthermore, 91.1% (95% CI 64.8–98.3) of the homeless or unstably housed patients had achieved SVR12, compared to 86.4% (95% CI 55.1–97.1) of those with stable homes. The pooled rates of SVR12 were 83.9% (95% CI 57.1–95.3) for patients with jobs and 84.5% (95% CI 75.9–90.4) for the unemployed patients. Finally, the SVR12 rates were 94.8% (95% CI 80.0–99.0) and 92.9% (95% CI 69.4–98.7) for patients in stable versus unstable relationships respectively. No significant differences were observed in other subgroup analyses.

### Subgroup analysis of LDV/SOF

We also conducted subgroup analysis on the basis of LDV/SOF regimen and treatment duration (Table 4). Among drug users that received LDV/SOF, 89.2% (95% CI 83.4–93.1) achieved SVR. In contrast, 90.4% (95% CI 83.6–94.5) of drug users treated with LDV/SOF ± RBV achieved SVR. Pooled rates of SVR12 were 88% (95% CI 70.7–95.7) at 8 weeks, 89.9% (95% CI 81.0–94.9) at 12 weeks, and 82.2% (95% CI 24.9–98.5) at 24 weeks. There were no significant differences between these subgroups.

**Table 4:** SVR12 of LDV/SOF by regimen and different treatment duration

Response	SVR12 (N = 711)		Heterogeneity		*P*^b^ value	Studies
	Total, n/N	Rate (95% CI)	*I*^2^ (%)	*P*^a^		
Overall	644/711	89.8 (85.9–92.7)	47.6	0.0336		12
By regimes						
LDV/SOF	436/484	89.2 (83.4–93.1)	55.6	0.7452	0.0274	9
LDV/SOF ± RBV	193/211	90.4 (83.6–94.5)	28.9			5
By different treatment duration						
8	93/101	88.0 (70.7–95.7)	46.2	0.8774	0.2627	5
12	74/82	89.9 (81.0–94.9)	0.0			3
24	16/18	82.2 (24.9–98.5)	58.2			2

^a^Test of heterogeneity

^b^Test for subgroup differences

### Different types of drugs used in drug users

Nine studies included data on the types of drugs used by the patients (Table 5). The number of heroin, cocaine, methamphetamine, buprenorphine, methadone, cannabis and benzodiazepines users were 129, 123, 58, 78, 313, 30 and 59 respectively. The pooled SVR12 rates were 51% (95% CI 35.9–65.4), 25.1% (95% CI 15.9–37.4), 58.2% (95% CI 41.9–72.9), 15% (95% CI 6.6–30.4), 55.7% (95% CI 29.2–79.3), 24.6% (95% CI 6.2–61.5) and 24.0% (95% CI 14.5–36.9) for the heroin, cocaine, methamphetamine, buprenorphine, methadone, cannabis and benzodiazepines users.

**Table 5:** Types of drugs used by the drug users

Drugs	Safety		Heterogeneity		Studies
	Total, n/N	Rate% (95% CI)	*I*^2^ (%)	*P*	
Heroine	129/287	51.0 (35.9–65.4)	80.4	0.0016	4
Cocaine	123/424	25.1 (15.9–37.4)	83.3	0.0005	4
Methamphetamine	58/103	58.2 (41.9–72.9)	56.9	0.1279	2
Buprenorphine	78/486	15.0 (6.6–30.4)	89.6	< 0.0001	5
Methadone	313/486	55.7 (29.2–79.3)	93.8	< 0.0001	5
Cannabis	30/122	24.6 (6.2–61.5)	92.1	0.0004	2
Benzodiazepines	59/272	24.0 (14.5–36.9)	76.9	0.0132	3

### Safety

Most of the included articles did not describe adverse events in detail, and only 8 had data on one or more indicators. As shown in Table 6, the cases with relapse, re-infection, virological failure, AEs, SAEs and discontinuation due to AEs were 4, 4, 11, 333, 17 and 2 respectively, and the pooled rates were 5.1% (95% CI 1.9–12.9), 5.7% (95% CI 2.2–14.2), 4.8% (95% CI 2.5–9.0), 77.8% (95% CI 64.5–87.1), 5.3% (95% CI 1.8–14.5) and 0.5% (95% CI 0.1–1.8). The most common AEs were fatigue (27.3%), nausea (16.1%), headache (13.9%), insomnia (5.1%), diarrhea (4.0%) and pain (2.7%). In patients with SAEs, one death occurred due to opioid drug overdose and one was related to asymptomatic neutropenia. For the two patients who discontinued treatment, detailed medical reasons were not provided.

**Table 6:** Rate of safety outcomes for drug users with HCV

Outcomes	Safety		Heterogeneity		Studies
	Total, n/N	Rate% (95% CI)	*I*^2^ (%)	*P*	
Virological relapse	4/81	5.1 (1.9–12.9)	0.0	0.5813	2
Virological reinfection	4/71	5.7 (2.2–14.2)	0.0	0.7930	2
Virological failure	11/250	4.8 (2.5–9.0)	5.3	0.3480	3
AEs	333/447	77.8 (64.5–87.1)	85.7	0.0001	4
SAEs	17/368	5.3 (1.8–14.5)	76.2	0.0056	4
Discontinuation due to AEs	2/453	0.5 (0.1–1.8)	0.0	0.8377	2

## Discussion

The pooled SVR12 rates of drug users treated with LDV/SOF was 89.8% (95% CI 85.9–92.7) in this meta-analysis, which was lower than that reported by Grebely et al. [35] (97%) for sofosbuvir/velpatasvir and glecaprevir/pibrentasvir(94.6%) [36] among drug users, and slightly higher than that achieved by elbasvir/grazoprevir (89.5%) [37] among drug users. Despite the high SVR12, sofosbuvir/velpatasvir was associated with high rates of AEs (83%), SAEs (7%), discontinuation due to AEs (1%) and virological relapse rates (25%) compared to LDV/SOF (AEs = 77.8%, SAEs = 5.3%, discontinuation due to AEs = 0.5% and virological relapse = 5.1%) [35, 38]. Therefore, LDV/SOF is a safe and relatively effective treatment option for treating HCV in drug users.

There are eight known genotypes of HCV, and nearly half (46%) of the global HCV genotypes are GT1, mainly in Europe, North America and Australia, followed by GT3 (30%) primarily distributed in South Asia, particularly the Indian sub-continent [39]. The pooled SVR12 rate was significantly higher in drug users infected with the GT1 (92.4%; 95% CI 88.6–95.0) compared to the total SVR12 rate (90.6%; 95% CI 87.1–93.3). SVR12 was only 80.9% (95% CI 65.8–90.3) in GT3 drug users treated with LDV/SOF. The efficacy of LDV/SOF against GT1 was also better compared to the other genotypes, which may be attributed to the small sample size of the latter. In addition, the SVR rate of paritaprevir, ritonavir, ombitasvir and dasabuvir with/out ribavirin was only 71% as opposed to the 91% achieved by boceprevir and telaprevir in drug users with GT1 virus [40, 41]. Thus, LDV/SOF is a suitable option for drug users with GT1 HCV. In addition to the HCV genotype, the use of LDV/SOF to treat HCV depends on whether the patient has liver cirrhosis [42]. Cirrhosis was mentioned in 8 of the 12 studies, of which two studies recruited subjects without cirrhosis, the exclusion criteria of two studies were decompensated cirrhosis, and the exclusion criterion for one study was that the Fib4 and fibrosis-cirrhosis index exceeded 3.25 and 1.25, respectively. However, there was no data on SVR12 of LDV/SOF in drug users with cirrhosis in all the included studies, and no related studies have been found. The next research needs to pay attention to this aspect.

In the 12 included studies, one study reported SVR12 rates of 95% (21/22) and 94% (45/48) for LDV/SOF + RBV and LDV/SOF respectively. Other studies had shown similar efficacies of LDV/SOF regimen with/out RBV, although inclusion of the latter increased the incidence of AEs and SAEs. Therefore, ribavirin is not recommended for treating HCV in combination with LDV and SOF [43–46]. Furthermore, our results indicate that treatment efficacy did not improve with the duration. While 12 weeks of LDV/SOF treatment had greater efficacy compared to the other time points, the least efficacy was observed after 24 weeks. In addition, a meta-analysis showed that 24 weeks LDV/SOF resulted in more SAEs in GT1 chronic hepatitis C patients compared to 8 weeks and 12 weeks treatments [20]. Thus, we recommend a 12-week treatment course of LDV/SOF for drug users with HCV infection.

In the ITT analysis, the SVR12 rate was 95% among people without a history of injecting drug use, 92% among PWID not receiving OAT [47]. A major issue with antiviral therapy for drug users is poor compliance [7]. The pooled SVR12 rates for drug users was lower than that of LDV/SOF-treated children (99%, 95% CI 94–100) [48], adolescents (98%, 95% CI 93–100) [49] and subjects aged 65 years or older (97%, 95% CI 96–98) [50] who were not drug users. It was also lower than the SVR12 rates in HCV patients with HBV (100%) [51], compensated liver disease (94%, 95% CI 84–99) [52] and kidney transplant (100%, 95% CI 94–100) [53] who were treated with LDV/SOF and were not drug users. Eight studies included in this meta-analysis had information regarding compliance of drug users taking LDV/SOF, of which 5 reported a compliance rate > 80% through self-reporting or collection of remaining drug doses. This was consistent with the 99% and 98% compliance rates of HCV-positive drug users through self-reporting and residual pill counting respectively as reported by Cunningham et al. [54]. However, the patients that received treatment twice a day had lower compliance. Two studies [55, 56] showed that false reporting and the loss of pills were associated with an overestimation of cure rates by self-reporting and residual pill counting. Among the remaining studies, one reported poor compliance, one stated that patients with addiction had a compliance rate of 62%, and the third achieved 39.2% and 49.9% compliance rates through non-direct and modified direct observation respectively. Macías et al. [47] showed that the compliance rate of active drug users was 79%, while the SVR12 rate of non-drug users was 95%. Taken together, drug users have relatively lower compliance to anti-viral treatment compared to non-users, which can be attributed to the high rate of loss during follow-up [57]. In this meta-analysis, the compliance among drug users treated with LDV/SOF was fair, as only 4% (29/727) of the cases were lost to follow-up.

There were still some limitations in our study that ought to be considered. First, six of the included studies analyzed effects of LDV/SOF in combination with other drugs. Furthermore, the basic information of drug users treated only with LDV/SOF was not available and the subgroup analysis of LDV/SOF was incomplete. Second, it was prone to include overlapping patients in Ref No 29 and 32. Third, methamphetamine and heroin were the main drugs used by drug addicts, but there was no information regarding how previous or recent use of different drugs affected SVR12. Fourth, the resistance of drug users to LDV/SOF was also unknown. Fifth, the sample size was small due to the few clinical studies published so far.

## Conclusion

This meta-analysis is the first to provide a comprehensive analysis of LDV/SOF treatment for drug users, with a total of 711 participants from 12 individual studies. Although there were still some limitations, the data showed that LDV/SOF is a safe and relatively effective for drug users with hepatitis C. Larger and better designed studies are needed to evaluate the effects of LDV-SOF and the treatment resistance in drug users. Future studies need to focus on providing effective measures to improve medication compliance among drug users and reduce loss during follow-up.

## Supplementary Information

**Additional file 1**. Literature search strategy.

**Additional file 2**. Quality assessment.

**Additional file 3**. Funnel plot, Egger’s test results and Sensitivity assessment.

**Additional file 4**. Prisma checklist.

## Data Availability

All data generated or analyzed in this study are included in this article and its supplementary information files.

## Abbreviations

LDV/SOF: Ledipasvir/sofosbuvir
CI: Confidence interval
RBV: Ribavirin
NA: Not applicable
OBV: Ombitasvir
PTV: Paritaprevir
r: Ritonavir
DSV: Dasabuvir
VEL: Velpatasvir
DCV: Daclatasvir
SMV: Simeprevir
VOX: Voxilaprevir
NR: Not reported
PEG: Pegylated interferon
AEs: Any adverse events
CAEs: Common adverse events
SAEs: Serious adverse events

## References

[1] Hwang JP, LoConte NK, Rice JP, Foxhall LE, Sturgis EM, Merrill JK, Torres HA, Bailey HH (2019). Oncologic implications of chronic hepatitis C virus infection. J Oncol Pract.

[2] WHO: global hepatitis report, 2017. 2017.

[3] Spearman CW, Dusheiko GM, Hellard M, Sonderup M (2019). Hepatitis C. Lancet.

[4] Pol S, Lagaye S (2019). The remarkable history of the hepatitis C virus. Genes Immun.

[5] Reed JR, Jordan AE, Perlman DC, Smith DJ, Hagan H (2016). The HCV care continuum among people who use drugs: protocol for a systematic review and meta-analysis. Syst Rev.

[6] Larney S, Grebely J, Hickman M, De Angelis D, Dore GJ, Degenhardt L (2015). Defining populations and injecting parameters among people who inject drugs: implications for the assessment of hepatitis C treatment programs. Int J Drug Policy.

[7] Ing Lorenzini K, Girardin F (2020). Direct-acting antiviral interactions with opioids, alcohol or illicit drugs of abuse in HCV-infected patients. Liver Int.

[8] Akiyama MJ, Cleland CM, Lizcano JA, Cherutich P, Kurth AE (2019). Prevalence, estimated incidence, risk behaviours, and genotypic distribution of hepatitis C virus among people who inject drugs accessing harm-reduction services in Kenya: a retrospective cohort study. Lancet Infect Dis.

[9] Higgs P, Sacks-Davis R, Gold J, Hellard M (2011). Barriers to receiving hepatitis C treatment for people who inject drugs: myths and evidence. Hepat Mon.

[10] Lusivika-Nzinga C, Fontaine H, Dorival C, Simony M, Pol S, Carrat F (2019). group AAHs: the dynamic effect of direct-acting antiviral treatments on the risk of hepatocellular carcinoma in patients with cirrhosis and chronic hepatitis C. J Viral Hepat.

[11] Afdhal N, Zeuzem S, Kwo P, Chojkier M, Gitlin N, Puoti M, Romero-Gomez M, Zarski JP, Agarwal K, Buggisch P (2014). Ledipasvir and sofosbuvir for untreated HCV genotype 1 infection. N Engl J Med.

[12] Floreani A (2015). Perspectives of fixed daily dose of sofosbuvir and ledipasvir for the treatment of chronic hepatitis C. Expert Opin Pharmacother.

[13] APASL consensus statements and recommendation on treatment of hepatitis C. http://apasl.info/guidelines/. 10.1007/s12072-016-9717-6 PMC5003907 27130427

[14] EASL recommendations on treatment of hepatitis C: final update of the series. https://easl.eu/publications/clinical-practice-guidelines/.

[15] Nouch S, Gallagher L, Erickson M, Elbaharia R, Zhang W, Wang L, Bacani N, Kason D, Kleban H, Knebel L (2018). Factors associated with lost to follow-up after hepatitis C treatment delivered by primary care teams in an inner-city multi-site program, Vancouver, Canada. Int J Drug Policy.

[16] Hajarizadeh B, Cunningham EB, Reid H, Law M, Dore GJ, Grebely J (2018). Direct-acting antiviral treatment for hepatitis C among people who use or inject drugs: a systematic review and meta-analysis. Lancet Gastroenterol Hepatol.

[17] Liberati A, Altman DG, Tetzlaff J, Mulrow C, Gotzsche PC, Ioannidis JP, Clarke M, Devereaux PJ, Kleijnen J, Moher D (2009). The PRISMA statement for reporting systematic reviews and meta-analyses of studies that evaluate healthcare interventions: explanation and elaboration. BMJ.

[18] Margulis AV, Pladevall M, Riera-Guardia N, Varas-Lorenzo C, Hazell L, Berkman ND, Viswanathan M, Perez-Gutthann S (2014). Quality assessment of observational studies in a drug-safety systematic review, comparison of two tools: the Newcastle-Ottawa Scale and the RTI item bank. Clin Epidemiol.

[19] Higgins JP, Altman DG, Gotzsche PC, Juni P, Moher D, Oxman AD, Savovic J, Schulz KF, Weeks L, Sterne JA (2011). The Cochrane Collaboration's tool for assessing risk of bias in randomised trials. BMJ.

[20] Tao T, Jiang X, Chen Y, Song Y (2017). Efficacy and safety of ledipasvir/sofosbuvir with and without ribavirin in patients with chronic hepatitis C virus genotype 1 infection: a meta-analysis. Int J Infect Dis.

[21] Hajarizadeh B, Cunningham EB, Valerio H, Martinello M, Law M, Janjua NZ, Midgard H, Dalgard O, Dillon J, Hickman M (2020). Hepatitis C reinfection after successful antiviral treatment among people who inject drugs: a meta-analysis. J Hepatol.

[22] Gane E, Lawitz E, Pugatch D, Papatheodoridis G, Brau N, Brown A, Pol S, Leroy V, Persico M, Moreno C (2017). Glecaprevir and pibrentasvir in patients with HCV and severe renal impairment. N Engl J Med.

[23] Gayam V, Tiongson B, Mandal AK, Garlapati P, Pan C, Mohanty S (2019). Real-world study of hepatitis C treatment with direct-acting antivirals in patients with drug abuse and opioid agonist therapy. Scand J Gastroenterol.

[24] Alimohammadi A, Magel T, Chu L, Yung R, Truong D, Holeksa J, Conway B (2019). A comparison of currently available direct acting antiviral HCV therapy in Canada: on the path to elimination. J Hepatol.

[25] Coffin PO, Santos GM, Behar E, Hern J, Walker J, Matheson T, Kinnard EN, Silvis J, Vittinghoff E, Fox R, Page K (2019). Randomized feasibility trial of directly observed versus unobserved hepatitis C treatment with ledipasvir-sofosbuvir among people who inject drugs. PLoS ONE.

[26] Schutz A, Moser S, Schwanke C, Schubert R, Luhn J, Gutic E, Lang T, Schleicher M, Haltmayer H, Gschwantler M (2018). Directly observed therapy of chronic hepatitis C with ledipasvir/sofosbuvir in people who inject drugs at risk of nonadherence to direct-acting antivirals. J Viral Hepat.

[27] Ovrehus ALH, Krarup H, Birkemose I, Holm DK, Mossner B, Ernst A, Christensen PB (2018). Four weeks of ledipasvir/sofosbuvir and ribavirin with or without pegylated interferon for chronic hepatitis C in non-cirrhotic people who inject drugs. A randomized trial. J Hepatol.

[28] Trabut JB, Barrault C, Charlot H, Carmona D, Bourdel A, Benslimane M, Francois M, Kini-Matondo W, Causse R, Roudot-Thoraval F, Hezode C (2018). Integrated care for the use of direct-acting antivirals in patients with chronic hepatitis C and substance use disorder. J Addict Med.

[29] Grebely J, Feld JJ, Wyles D, Sulkowski M, Ni L, Llewellyn J, Mir HM, Sajed N, Stamm LM, Hyland RH (2018). Sofosbuvir-based direct-acting antiviral therapies for HCV in people receiving opioid substitution therapy: an analysis of phase 3 studies. Open Forum Infect Dis.

[30] Morris L, Smirnov A, Kvassay A, Leslie E, Kavanagh R, Alexander N, Davey G, Williams O, Gilks C, Najman J (2017). Initial outcomes of integrated community-based hepatitis C treatment for people who inject drugs: findings from the Queensland Injectors' Health Network. Int J Drug Policy.

[31] Read P, Lothian R, Chronister K, Gilliver R, Kearley J, Dore GJ, van Beek I (2017). Delivering direct acting antiviral therapy for hepatitis C to highly marginalised and current drug injecting populations in a targeted primary health care setting. Int J Drug Policy.

[32] Grebely J, Mauss S, Brown A, Bronowicki JP, Puoti M, Wyles D, Natha M, Zhu Y, Yang J, Kreter B (2016). Efficacy and safety of ledipasvir/sofosbuvir with and without ribavirin in patients with chronic HCV genotype 1 infection receiving opioid substitution therapy: analysis of phase 3 ION trials. Clin Infect Dis.

[33] Akiyama M, Lizcano J, Musyoki H, Cherutich P, Kurth A (2020). Hepatitis C treatment outcomes among people who inject drugs accessing harm reduction services in Kenya. J Hepatol.

[34] Akiyama MJ, Norton BL, Arnsten JH, Agyemang L, Heo M, Litwin AH (2019). Intensive models of hepatitis C care for people who inject drugs receiving opioid agonist therapy: a randomized controlled trial. Ann Intern Med.

[35] Grebely J, Dalgard O, Conway B, Cunningham EB, Bruggmann P, Hajarizadeh B, Amin J, Bruneau J, Hellard M, Litwin AH (2018). Sofosbuvir and velpatasvir for hepatitis C virus infection in people with recent injection drug use (SIMPLIFY): an open-label, single-arm, phase 4, multicentre trial. Lancet Gastroenterol Hepatol.

[36] Schmidbauer C, Schubert R, Schütz A, Schwanke C, Luhn J, Gutic E, Pirker R, Lang T, Reiberger T, Haltmayer H, Gschwantler M (2020). Directly observed therapy for HCV with glecaprevir/pibrentasvir alongside opioid substitution in people who inject drugs-First real world data from Austria. PLoS ONE.

[37] Dore GJ, Altice F, Litwin AH, Dalgard O, Gane EJ, Shibolet O, Luetkemeyer A, Nahass R, Peng CY, Conway B (2016). Elbasvir-grazoprevir to treat hepatitis C virus infection in persons receiving opioid agonist therapy: a randomized trial. Ann Intern Med.

[38] Janjua NZ, Darvishian M, Wong S, Yu A, Rossi C, Ramji A, Yoshida EM, Butt ZA, Samji H, Chong M (2019). Effectiveness of ledipasvir/sofosbuvir and sofosbuvir/velpatasvir in people who inject drugs and/or those in opioid agonist therapy. Hepatol Commun.

[39] Tsukiyama-Kohara K, Kohara M (2017). Hepatitis C virus: viral quasispecies and genotypes. Int J Mol Sci.

[40] Grebely J, Conway B, Cunningham EB, Fraser C, Moriggia A, Gane E, Stedman C, Cooper C, Castro E, Schmid P (2018). Paritaprevir, ritonavir, ombitasvir, and dasabuvir with and without ribavirin in people with HCV genotype 1 and recent injecting drug use or receiving opioid substitution therapy. Int J Drug Policy.

[41] Arain A, Bourgeois S, de Galocsy C, Henrion J, Deltenre P, d'Heygere F, George C, Bastens B, Van Overbeke L, Verrando R (2016). Belgian experience with triple therapy with boceprevir and telaprevir in genotype 1 infected patients who inject drugs. J Med Virol.

[42] Margusino-Framiñán L, Cid-Silva P, Giménez-Arufe V, Mondelo-García C, Fernández-Oliveira C, Mena-de-Cea Á, Martín-Herranz I, Castro-Iglesias Á (2021). Influence of drug-drug interactions on effectiveness and safety of direct-acting antivirals against hepatitis C virus. Eur J Hosp Pharm.

[43] Thu Thuy PT, Bunchorntavakul C, Tan Dat H, Palecki J, Reddy KR (2018). Sofosbuvir-ledipasvir with or without ribavirin for chronic hepatitis C genotype-1 and 6: real-world experience in Vietnam. Antivir Ther.

[44] Scott LJ (2018). Ledipasvir/sofosbuvir: a review in chronic hepatitis C. Drugs.

[45] Gane EJ, Hyland RH, An D, Svarovskaia E, Pang PS, Brainard D, Stedman CA (2015). Efficacy of ledipasvir and sofosbuvir, with or without ribavirin, for 12 weeks in patients with HCV genotype 3 or 6 infection. Gastroenterology.

[46] Terrault NA, Zeuzem S, Di Bisceglie AM, Lim JK, Pockros PJ, Frazier LM, Kuo A, Lok AS, Shiffman ML, Ben Ari Z (2016). Effectiveness of ledipasvir-sofosbuvir combination in patients with hepatitis C virus infection and factors associated with sustained virologic response. Gastroenterology.

[47] Macias J, Morano LE, Tellez F, Granados R, Rivero-Juarez A, Palacios R, Rios M, Merino D, Perez-Perez M, Collado A (2019). Response to direct-acting antiviral therapy among ongoing drug users and people receiving opioid substitution therapy. J Hepatol.

[48] Murray KF, Balistreri WF, Bansal S, Whitworth S, Evans HM, Gonzalez-Peralta RP, Wen J, Massetto B, Kersey K, Shao J (2018). Safety and efficacy of ledipasvir-sofosbuvir with or without ribavirin for chronic hepatitis c in children ages 6–11. Hepatology.

[49] Balistreri WF, Murray KF, Rosenthal P, Bansal S, Lin CH, Kersey K, Massetto B, Zhu Y, Kanwar B, German P (2017). The safety and effectiveness of ledipasvir-sofosbuvir in adolescents 12–17 years old with hepatitis C virus genotype 1 infection. Hepatology.

[50] Saab S, Park SH, Mizokami M, Omata M, Mangia A, Eggleton E, Zhu Y, Knox SJ, Pang P, Subramanian M (2016). Safety and efficacy of ledipasvir/sofosbuvir for the treatment of genotype 1 hepatitis C in subjects aged 65 years or older. Hepatology.

[51] Liu CJ, Chuang WL, Sheen IS, Wang HY, Chen CY, Tseng KC, Chang TT, Massetto B, Yang JC, Yun C (2018). Efficacy of ledipasvir and sofosbuvir treatment of HCV infection in patients coinfected with HBV. Gastroenterology.

[52] Moser S, Kozbial K, Laferl H, Schutz A, Reiberger T, Schwabl P, Gutic E, Schwanke C, Schubert R, Luhn J (2018). Efficacy of ledipasvir/sofosbuvir plus ribavirin for 12 weeks in patients with chronic hepatitis C genotype 3 and compensated liver disease. Eur J Gastroenterol Hepatol.

[53] Colombo M, Aghemo A, Liu H, Zhang J, Dvory-Sobol H, Hyland R, Yun C, Massetto B, Brainard DM, McHutchison JG (2017). Treatment with ledipasvir-sofosbuvir for 12 or 24 weeks in kidney transplant recipients with chronic hepatitis C virus genotype 1 or 4 infection: a randomized trial. Ann Intern Med.

[54] Cunningham EB, Hajarizadeh B, Amin J, Litwin AH, Gane E, Cooper C, Lacombe K, Hellard M, Read P, Powis J (2019). Adherence to once-daily and twice-daily direct acting antiviral therapy for hepatitis C infection among people with recent injection drug use or current opioid agonist therapy. Clin Infect Dis.

[55] Cunningham EB, Hajarizadeh B, Dalgard O, Amin J, Hellard M, Foster GR, Bruggmann P, Conway B, Backmund M, Robaeys G (2017). Adherence to response-guided pegylated interferon and ribavirin for people who inject drugs with hepatitis C virus genotype 2/3 infection: the ACTIVATE study. BMC Infect Dis.

[56] Mason K, Dodd Z, Guyton M, Tookey P, Lettner B, Matelski J, Sockalingam S, Altenberg J, Powis J (2017). Understanding real-world adherence in the directly acting antiviral era: a prospective evaluation of adherence among people with a history of drug use at a community-based program in Toronto, Canada. Int J Drug Policy.

[57] Read P, Gilliver R, Kearley J, Lothian R, Cunningham EB, Chronister KJ, Dore GJ (2019). Treatment adherence and support for people who inject drugs taking direct-acting antiviral therapy for hepatitis C infection. J Viral Hepat.

