# Supplementary material for: Efficacy and safety of ledipasvir/sofosbuvir for hepatitis C among drug users: a systematic review and meta-analysis
Source: Virol J. 2021 Jul 27;18:156. doi: 10.1186/s12985-021-01625-w (PMC8314543; doi:10.1186/s12985-021-01625-w)

***Literature Search Strategy***

1. **Cochrane-84**

Search Name: 12

Comment:

ID Search Hits

#1 MeSH descriptor: [Hepatitis C] explode all trees 3326

#2 (Parenterally-Transmitted Non-A, Non-B Hepatitis):ti,ab,kw OR (Parenterally Transmitted Non A, Non B Hepatitis):ti,ab,kw OR (PT-NANBH):ti,ab,kw OR (Hepatitis, Viral, Non-A, Non-B, Parenterally-Transmitted):ti,ab,kw OR (Hepatitis C virus):ti,ab,kw OR (Hepatitis C virus):ti,ab,kw OR (Hepatitis C viruses):ti,ab,kw 5256

#3 #1 or #2 6581

#4 (Drug User):ti,ab,kw OR (User, Drug):ti,ab,kw OR (Users, Drug):ti,ab,kw OR (Drug Abusers):ti,ab,kw OR (Abuser, Drug):ti,ab,kw OR (Abusers, Drug):ti,ab,kw OR (Drug Abuser):ti,ab,kw OR (Drug Addicts):ti,ab,kw OR (Injection drug use):ti,ab,kw OR (people who inject drugs):ti,ab,kw OR (people who use drugs):ti,ab,kw OR (IDU):ti,ab,kw OR (PWID):ti,ab,kw OR (inject drug):ti,ab,kw OR (injecting drug):ti,ab,kw OR (drug inject):ti,ab,kw OR (drug use):ti,ab,kw OR (opioid substitution):ti,ab,kw OR (OST):ti,ab,kw OR (opioid agonist):ti,ab,kw OR (OAT):ti,ab,kw OR (methadone therapy):ti,ab,kw OR (methadone treat):ti,ab,kw OR (MMT):ti,ab,kw OR (IDUS):ti,ab,kw 267372

#5 (ledipasvir, sofosbuvir drug combination):ti,ab,kw OR (ledipasvir-sofosbuvir):ti,ab,kw OR (Harvoni):ti,ab,kw OR (sofosbuvir-ledipasvir):ti,ab,kw OR (ledipasvir and sofosbuvir):ti,ab,kw OR (sofosbuvir and ledipasvir):ti,ab,kw OR (sofosbuvir plus ledipasvir):ti,ab,kw OR (ledipasvir plus sofosbuvir):ti,ab,kw 362

#6 #3 and #4 and #5 84

1. PUBMED-31

| Search number | Query | Sort By | Filters | Search Details | Results | Time |
| --- | --- | --- | --- | --- | --- | --- |
| 10 | ((("Hepatitis C"[Mesh]) OR (((((((Parenterally-Transmitted Non-A, Non-B Hepatitis[Title/Abstract]) OR (Parenterally Transmitted Non A, Non B Hepatitis[Title/Abstract])) OR (PT-NANBH[Title/Abstract])) OR (Hepatitis, Viral, Non-A, Non-B, Parenterally-Transmitted[Title/Abstract])) OR (HCV[Title/Abstract])) OR (Hepatitis C virus[Title/Abstract])) OR (Hepatitis C viruses[Title/Abstract]))) AND (((("ledipasvir, sofosbuvir drug combination" [Supplementary Concept]) OR (((((((((ledipasvir - sofosbuvir[Title/Abstract]) OR Harvoni[Title/Abstract]) OR sofosbuvir-ledipasvir[Title/Abstract]) OR ledipasvir/sofosbuvir[Title/Abstract]) OR sofosbuvir/ledipasvir[Title/Abstract]) OR (sofosbuvir[Title/Abstract] AND ledipasvir[Title/Abstract])) OR (ledipasvir[Title/Abstract] AND sofosbuvir[Title/Abstract])) OR ledipasvir puls sofosbuvir[Title/Abstract]) OR sofosbuvir puls ledipasvir[Title/Abstract]))))) ((("Drug Users"[Mesh]) OR ((((((((((((((((((((((((Drug User[Title/Abstract]) OR (User, Drug[Title/Abstract])) OR (Users, Drug[Title/Abstract])) OR (Drug Abusers[Title/Abstract])) OR (Abuser, Drug[Title/Abstract])) OR (Abusers, Drug[Title/Abstract])) OR (Drug Abuser[Title/Abstract])) OR (Drug Addicts[Title/Abstract])) OR (Injection drug use[Title/Abstract])) OR (people who inject drugs[Title/Abstract])) OR (IDU[Title/Abstract])) OR (PWID[Title/Abstract])) OR (inject drug[Title/Abstract])) OR (injecting drug[Title/Abstract])) OR (drug inject[Title/Abstract])) OR (drug use[Title/Abstract])) OR (Injection drug user[Title/Abstract])) OR (opioid substitution[Title/Abstract])) OR (OST[Title/Abstract])) OR (opioid agonist[Title/Abstract])) OR (OAT[Title/Abstract])) OR (methadone therapy[Title/Abstract])) OR (methadone treat[Title/Abstract])) OR (MMT[Title/Abstract])) OR (IDUS[Title/Abstract]))) | | | (("Hepatitis C"[MeSH Terms] OR ("parenterally transmitted non a non b hepatitis"[Title/Abstract] OR "parenterally transmitted non a non b hepatitis"[Title/Abstract] OR "PT-NANBH"[Title/Abstract] OR ((("hepatitis a"[MeSH Terms] OR "hepatitis a"[All Fields] OR ("Hepatitis"[All Fields] AND "viral"[All Fields]) OR "hepatitis viral"[All Fields]) AND "Non-A"[All Fields] AND "Non-B"[All Fields]) AND "Parenterally-Transmitted"[Title/Abstract]) OR "HCV"[Title/Abstract] OR "hepatitis c virus"[Title/Abstract] OR "hepatitis c viruses"[Title/Abstract])) AND ("ledipasvir sofosbuvir drug combination"[Supplementary Concept] OR ("ledipasvir sofosbuvir"[Title/Abstract] OR "Harvoni"[Title/Abstract] OR "sofosbuvir-ledipasvir"[Title/Abstract] OR "ledipasvir sofosbuvir"[Title/Abstract] OR "sofosbuvir-ledipasvir"[Title/Abstract] OR ("sofosbuvir"[Title/Abstract] AND "ledipasvir"[Title/Abstract]) OR ("ledipasvir"[Title/Abstract] AND "sofosbuvir"[Title/Abstract]) OR ((("ledipasvir"[Supplementary Concept] OR "ledipasvir"[All Fields]) AND "puls"[All Fields]) AND "sofosbuvir"[Title/Abstract]) OR ((("sofosbuvir"[MeSH Terms] OR "sofosbuvir"[All Fields]) AND "puls"[All Fields]) AND "ledipasvir"[Title/Abstract])))) AND ("Drug Users"[MeSH Terms] OR ("drug user"[Title/Abstract] OR "user drug"[Title/Abstract] OR "users drug"[Title/Abstract] OR "drug abusers"[Title/Abstract] OR "abuser drug"[Title/Abstract] OR "abusers drug"[Title/Abstract] OR "drug abuser"[Title/Abstract] OR "drug addicts"[Title/Abstract] OR "injection drug use"[Title/Abstract] OR "people who inject drugs"[Title/Abstract] OR "IDU"[Title/Abstract] OR "PWID"[Title/Abstract] OR "inject drug"[Title/Abstract] OR "injecting drug"[Title/Abstract] OR ("Drug"[All Fields] AND "inject"[Title/Abstract]) OR "drug use"[Title/Abstract] OR "injection drug user"[Title/Abstract] OR "opioid substitution"[Title/Abstract] OR "OST"[Title/Abstract] OR "opioid agonist"[Title/Abstract] OR "OAT"[Title/Abstract] OR "methadone therapy"[Title/Abstract] OR (("methadon"[All Fields] OR "methadone"[MeSH Terms] OR "methadone"[All Fields] OR "methadone s"[All Fields]) AND "treat"[Title/Abstract]) OR "MMT"[Title/Abstract]) OR "IDUS"[Title/Abstract]) | 31 | 2:25:08 |
| 9 | (("Drug Users"[Mesh]) OR ((((((((((((((((((((((((Drug User[Title/Abstract]) OR (User, Drug[Title/Abstract])) OR (Users, Drug[Title/Abstract])) OR (Drug Abusers[Title/Abstract])) OR (Abuser, Drug[Title/Abstract])) OR (Abusers, Drug[Title/Abstract])) OR (Drug Abuser[Title/Abstract])) OR (Drug Addicts[Title/Abstract])) OR (Injection drug use[Title/Abstract])) OR (people who inject drugs[Title/Abstract])) OR (IDU[Title/Abstract])) OR (PWID[Title/Abstract])) OR (inject drug[Title/Abstract])) OR (injecting drug[Title/Abstract])) OR (drug inject[Title/Abstract])) OR (drug use[Title/Abstract])) OR (Injection drug user[Title/Abstract])) OR (opioid substitution[Title/Abstract])) OR (OST[Title/Abstract])) OR (opioid agonist[Title/Abstract])) OR (OAT[Title/Abstract])) OR (methadone therapy[Title/Abstract])) OR (methadone treat[Title/Abstract])) OR (MMT[Title/Abstract])) OR (IDUS[Title/Abstract])) | | | "Drug Users"[MeSH Terms] OR ("drug user"[Title/Abstract] OR "user drug"[Title/Abstract] OR "users drug"[Title/Abstract] OR "drug abusers"[Title/Abstract] OR "abuser drug"[Title/Abstract] OR "abusers drug"[Title/Abstract] OR "drug abuser"[Title/Abstract] OR "drug addicts"[Title/Abstract] OR "injection drug use"[Title/Abstract] OR "people who inject drugs"[Title/Abstract] OR "IDU"[Title/Abstract] OR "PWID"[Title/Abstract] OR "inject drug"[Title/Abstract] OR "injecting drug"[Title/Abstract] OR ("Drug"[All Fields] AND "inject"[Title/Abstract]) OR "drug use"[Title/Abstract] OR "injection drug user"[Title/Abstract] OR "opioid substitution"[Title/Abstract] OR "OST"[Title/Abstract] OR "opioid agonist"[Title/Abstract] OR "OAT"[Title/Abstract] OR "methadone therapy"[Title/Abstract] OR (("methadon"[All Fields] OR "methadone"[MeSH Terms] OR "methadone"[All Fields] OR "methadone s"[All Fields]) AND "treat"[Title/Abstract]) OR "MMT"[Title/Abstract]) OR "IDUS"[Title/Abstract] | 81,329 | 2:23:55 |
| 5 | ((("ledipasvir, sofosbuvir drug combination" [Supplementary Concept]) OR (((((((((ledipasvir - sofosbuvir[Title/Abstract]) OR Harvoni[Title/Abstract]) OR sofosbuvir-ledipasvir[Title/Abstract]) OR ledipasvir/sofosbuvir[Title/Abstract]) OR sofosbuvir/ledipasvir[Title/Abstract]) OR (sofosbuvir[Title/Abstract] AND ledipasvir[Title/Abstract])) OR (ledipasvir[Title/Abstract] AND sofosbuvir[Title/Abstract])) OR ledipasvir puls sofosbuvir[Title/Abstract]) OR sofosbuvir puls ledipasvir[Title/Abstract]))) | | | "ledipasvir sofosbuvir drug combination"[Supplementary Concept] OR ("ledipasvir sofosbuvir"[Title/Abstract] OR "Harvoni"[Title/Abstract] OR "sofosbuvir-ledipasvir"[Title/Abstract] OR "ledipasvir sofosbuvir"[Title/Abstract] OR "sofosbuvir-ledipasvir"[Title/Abstract] OR ("sofosbuvir"[Title/Abstract] AND "ledipasvir"[Title/Abstract]) OR ("ledipasvir"[Title/Abstract] AND "sofosbuvir"[Title/Abstract]) OR ((("ledipasvir"[Supplementary Concept] OR "ledipasvir"[All Fields]) AND "puls"[All Fields]) AND "sofosbuvir"[Title/Abstract]) OR ((("sofosbuvir"[MeSH Terms] OR "sofosbuvir"[All Fields]) AND "puls"[All Fields]) AND "ledipasvir"[Title/Abstract])) | 1,094 | 1:55:17 |
| 3 | ("Hepatitis C"[Mesh]) OR (((((((Parenterally-Transmitted Non-A, Non-B Hepatitis[Title/Abstract]) OR (Parenterally Transmitted Non A, Non B Hepatitis[Title/Abstract])) OR (PT-NANBH[Title/Abstract])) OR (Hepatitis, Viral, Non-A, Non-B, Parenterally-Transmitted[Title/Abstract])) OR (HCV[Title/Abstract])) OR (Hepatitis C virus[Title/Abstract])) OR (Hepatitis C viruses[Title/Abstract])) | | | "Hepatitis C"[MeSH Terms] OR ("parenterally transmitted non a non b hepatitis"[Title/Abstract] OR "parenterally transmitted non a non b hepatitis"[Title/Abstract] OR "PT-NANBH"[Title/Abstract] OR ((("hepatitis a"[MeSH Terms] OR "hepatitis a"[All Fields] OR ("Hepatitis"[All Fields] AND "viral"[All Fields]) OR "hepatitis viral"[All Fields]) AND "Non-A"[All Fields] AND "Non-B"[All Fields]) AND "Parenterally-Transmitted"[Title/Abstract]) OR "HCV"[Title/Abstract] OR "hepatitis c virus"[Title/Abstract] OR "hepatitis c viruses"[Title/Abstract]) | 92,673 | 1:53:08 |
| 2 | ((((((Parenterally-Transmitted Non-A, Non-B Hepatitis[Title/Abstract]) OR (Parenterally Transmitted Non A, Non B Hepatitis[Title/Abstract])) OR (PT-NANBH[Title/Abstract])) OR (Hepatitis, Viral, Non-A, Non-B, Parenterally-Transmitted[Title/Abstract])) OR (HCV[Title/Abstract])) OR (Hepatitis C virus[Title/Abstract])) OR (Hepatitis C viruses[Title/Abstract]) | | | "parenterally transmitted non a non b hepatitis"[Title/Abstract] OR "parenterally transmitted non a non b hepatitis"[Title/Abstract] OR "PT-NANBH"[Title/Abstract] OR ((("hepatitis a"[MeSH Terms] OR "hepatitis a"[All Fields] OR ("Hepatitis"[All Fields] AND "viral"[All Fields]) OR "hepatitis viral"[All Fields]) AND "Non-A"[All Fields] AND "Non-B"[All Fields]) AND "Parenterally-Transmitted"[Title/Abstract]) OR "HCV"[Title/Abstract] OR "hepatitis c virus"[Title/Abstract] OR "hepatitis c viruses"[Title/Abstract] | 73,555 | 1:52:39 |
| 1 | "Hepatitis C"[Mesh] | Most Recent | | "Hepatitis C"[MeSH Terms] | 65,628 | 1:49:59 |

1. **Embase-198**

Embase

Session Results

.......................................................

No. Query Results Results Date

#10. #3 AND #6 AND #9 198 18 Apr 2021

#9. #7 OR #8 3,725 18 Apr 2021

#8. 'ledipasvir, sofosbuvir drug combination':ab,ti 2,315 18 Apr 2021

OR 'ledipasvir - sofosbuvir':ab,ti OR

'harvoni':ab,ti OR 'sofosbuvir-ledipasvir':ab,ti

OR 'ledipasvir /sofosbuvir':ab,ti OR 'sofosbuvir

/ledipasvir':ab,ti OR 'sofosbuvir and

ledipasvir':ab,ti OR 'ledipasvir and

sofosbuvir':ab,ti OR 'sofosbuvir puls

ledipasvir':ab,ti

#7. 'ledipasvir plus sofosbuvir'/exp 3,127 18 Apr 2021

#6. #4 OR #5 113,280 18 Apr 2021

#5. 'drug user':ab,ti OR 'user, drug':ab,ti OR 112,770 18 Apr 2021

'users, drug':ab,ti OR 'drug abusers':ab,ti OR

'abuser, drug':ab,ti OR 'abusers, drug':ab,ti OR

'drug abuser':ab,ti OR 'drug addicts':ab,ti OR

'injection drug use':ab,ti OR 'people who inject

drugs':ab,ti OR 'idu':ab,ti OR 'pwid':ab,ti OR

'inject drug':ab,ti OR 'injecting drug':ab,ti OR

'drug inject':ab,ti OR 'drug use':ab,ti OR 'drug

users':ab,ti OR 'opioid substitution':ab,ti OR

'ost':ab,ti OR 'opioid agonist':ab,ti OR

'oat':ab,ti OR 'methadone therapy':ab,ti OR

'methadone treat':ab,ti OR 'mmt':ab,ti OR

'idus':ab,ti

#4. 'injection drug user'/exp 2,984 18 Apr 2021

#3. #1 OR #2 166,891 18 Apr 2021

#2. 'parenterally-transmitted non-a, non-b 117,249 18 Apr 2021

hepatitis':ab,ti OR 'parenterally transmitted non

a, non b hepatitis':ab,ti OR 'pt-nanbh':ab,ti OR

'hepatitis, viral, non-a, non-b,

parenterally-transmitted':ab,ti OR 'hcv':ab,ti OR

'hepatitis c virus':ab,ti OR 'hepatitis c

viruses':ab,ti

#1. 'hepatitis c'/exp 121,460 18 Apr 2021

.......................................................

1. **Web of science-1407**


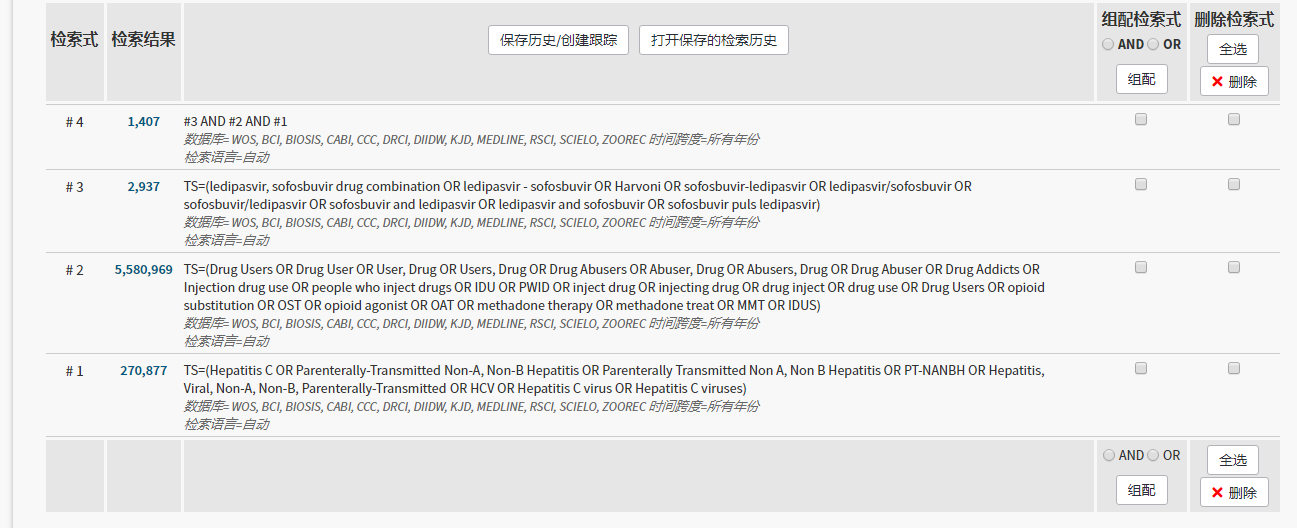

Supplement: Supplementary file 1 — Additional file 1. Literature search strategy. [file 12985_2021_1625_MOESM1_ESM.doc]
