# Supplementary material for: Efficacy and safety of ledipasvir/sofosbuvir for hepatitis C among drug users: a systematic review and meta-analysis
Source: Virol J. 2021 Jul 27;18:156. doi: 10.1186/s12985-021-01625-w (PMC8314543; doi:10.1186/s12985-021-01625-w)

***Quality assessment***

Table 1. Quality assessment of NOS.

| Study | Selection | Comparability | Outcome | Quality Score |
| --- | --- | --- | --- | --- |
| Vijay Gayam (2019) | ★★★★ | ★★ | ★★★ | 9 |
| A. ALIMOHAMMADI (2019) | ★★★ | ★ | ★★ | 6 |
| Angelika Schütz (2018) | ★★ | - | ★★★ | 5 |
| Jean-Baptiste Trabut (2018) | ★★★ | ★★ | ★★★ | 8 |
| Leith Morris (2017) | ★★★ | ★ | ★★★ | 7 |
| Phillip Read (2017) | ★★★ | ★ | ★★★ | 7 |

NOS, Newcastle-Ottawa quality assessment scale.

Fig. 1. Assessment of the quality of the studies included in the comprehensive analysis.


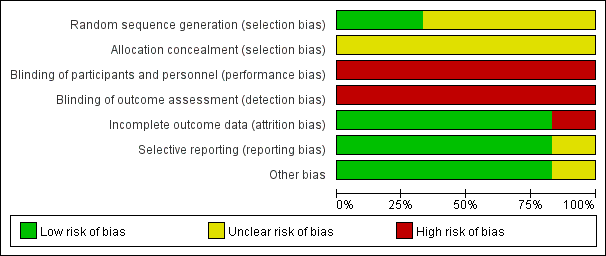


Fig. 2. Assessment of the quality of the studies included in the comprehensive analysis.


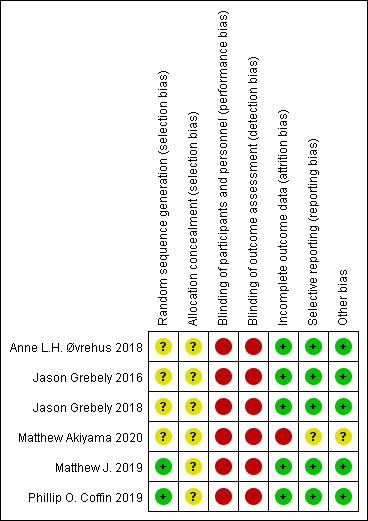

Supplement: Supplementary file 2 — Additional file 2. Quality assessment. [file 12985_2021_1625_MOESM2_ESM.doc]
