# Supplementary material for: Efficacy and safety of ledipasvir/sofosbuvir for hepatitis C among drug users: a systematic review and meta-analysis
Source: Virol J. 2021 Jul 27;18:156. doi: 10.1186/s12985-021-01625-w (PMC8314543; doi:10.1186/s12985-021-01625-w)

***Funnel plot, Egger’s test results and Sensitivity assessment***

Fig. 1. Funnel plot for the evaluation of publication bias of the whole study.


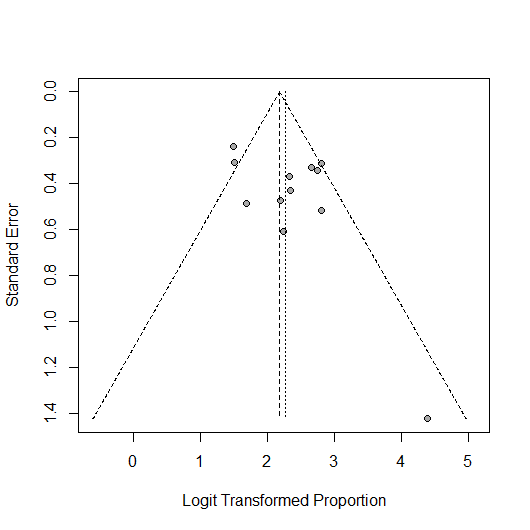


Fig. 2. Egger’s funnel plot for the evaluation of publication bias of the whole study.


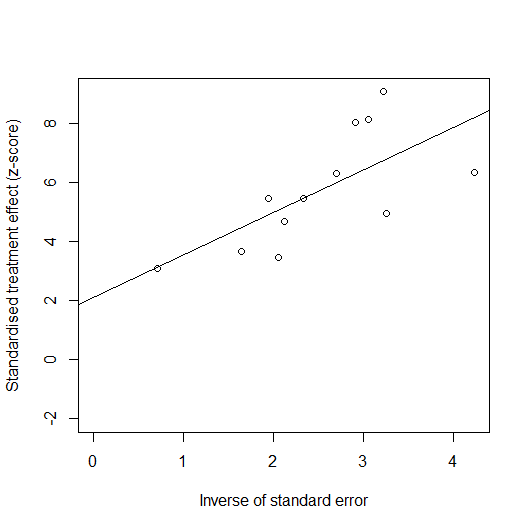


Fig. 3. Funnel plot for the evaluation of publication bias of the LDV/SOF.


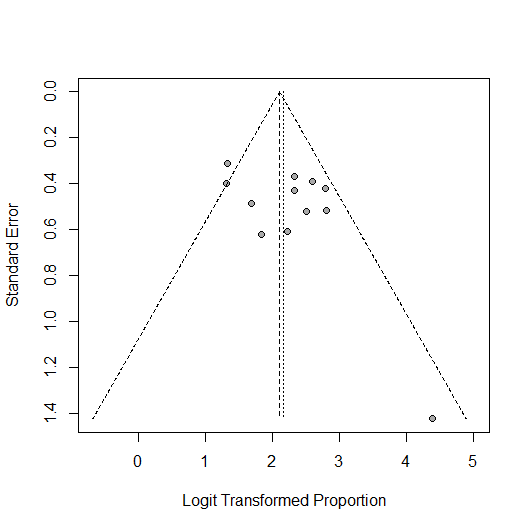


Fig. 4. Egger’s funnel plot for the evaluation of publication bias of the LDV/SOF.


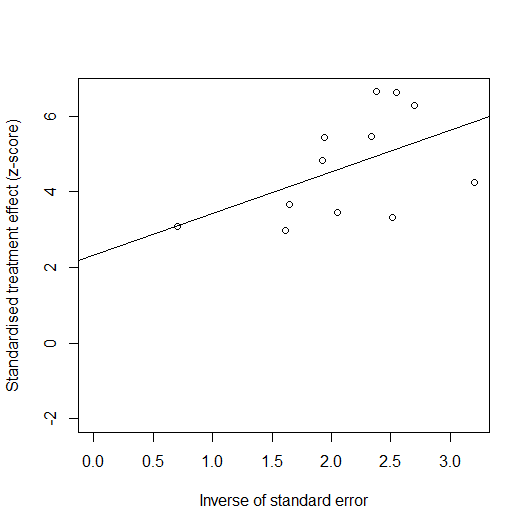


Fig. 5. The sensitivity analysis of SVR12 rate.


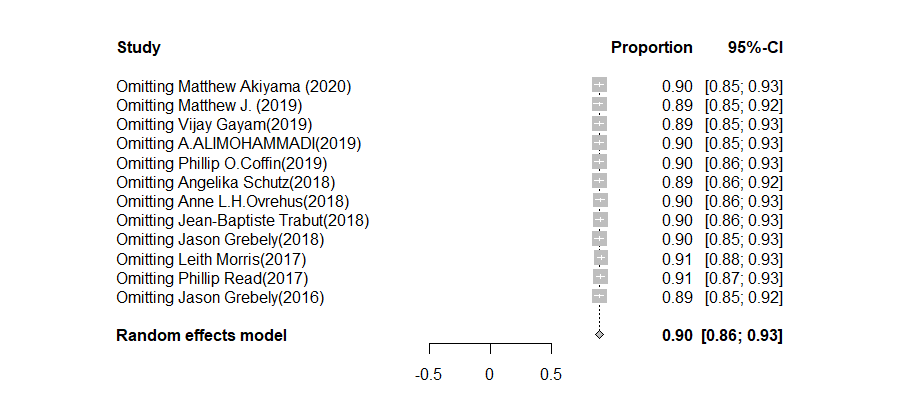

Supplement: Supplementary file 3 — Additional file 3. Funnel plot, Egger’s test results and Sensitivity assessment. [file 12985_2021_1625_MOESM3_ESM.doc]
